# Hypocalcemia and massive pulmonary embolism in SLE patient with COVID‐19 infection: A case report

**DOI:** 10.1002/ccr3.5115

**Published:** 2021-11-19

**Authors:** Mohammed Yousif Elnaeem Yousif, Moh.Mah.Fadel Allah Eljack, Mohammed Alfatih, Mohammed Abdulkarim, Abdirizak Ibrahim mohamed, Khabab Abbasher Hussien Mohamed Ahmed, Mazin S. Hassan Haroun

**Affiliations:** ^1^ Internal Medicine University of Gezira Khartoum Sudan; ^2^ Wad Medani Isolation Centers Khartoum Sudan; ^3^ Medani Heart Diseases and Surgery Center University of Bakht Alruda Khartoum Sudan; ^4^ Alzaiem Alazhari University Khartoum Sudan; ^5^ Medical Doctor at Kiambu level 5 Hospital University of Bakht Alruda Nairobi Kenya; ^6^ Faculty of Medicine University of Khartoum Khartoum Sudan

**Keywords:** COVID‐19, hypocalcemia, pulmonary embolism, SLE, Wad‐Madani

## Abstract

A 16‐year‐old female patient presented to our ED with fever and coughing of blood for 3 days. She is known to have SLE for 5 months and takes oral prednisone. She was tested positive for COVID‐19. She developed hypocalcemia with clinically diagnosed massive pulmonary embolism. She was treated with heparin and recovered.

## Graphical abstract

In this study, we report a case of a 16‐year‐old female patient who is a known case of systemic lupus erythematosus (SLE) for 5 months and is on steroids presenting with hypocalcemia and massive pulmonary embolism diagnosed clinically while she has COVID.

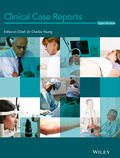

## 1 INTRODUCTION

Systemic lupus erythematosus (SLE) is an autoimmune disease that is characterized with autoantibodies production with on and off clinical course. It can affect any age or gender, but it has a particular tendency to affect young females, with a female‐to‐male ratio of about 9:1.^1^

SLE can affect almost every organ system with a diverse scale of manifestations. The disease severity can vary from a very mild illness to a systemic life‐threatening illness.^2^, ^3^, ^4^ Although manifestations can affect almost every system in the body, cardiac and lung manifestations have a significant impact on patients’ everyday life and outcome. Respiratory affection can affect the lung, pleura, or lung vasculature with various degrees of affection from an asymptomatic illness to severe respiratory compromise.

## 2 CASE HISTORY/EXAMINATION

A 16‐year‐old female patient has presented to our accidents and emergency department complaining of fever and coughing of blood for 3 days. Her condition started 3 days prior to admission with a gradual‐onset, high‐grade fever that was associated with rigors. She also had a productive cough of red bloody sputum. There was no associated chest pain, shortness of breath, syncopal attack, nor lower limb edema.

On review of her systems including GI, GU, and CNS, she reported burning epigastric pain with no abdominal distension, nausea, vomiting, diarrhea, or constipation. There was no weight loss nor change in her appetite.

When it comes to her past medical history, she reported that she had been diagnosed with systemic lupus erythematosus (SLE) 5 months ago when she sought medical advice regarding recurrent facial rashes and small joints pain. Her SLE has been immunologically confirmed using anti‐double stranded DNA antibodies (100 IU/ml) and ANA factor (400 IU/ml) for which she currently takes prednisone 5 mg once daily and hydroxychloroquine tabs 200 mg tabs twice daily with good adherence to treatment and regular follow‐up since then. Apart from SLE, she reported no DM, HTN, or any other coagulation or autoimmune diseases and she has never been hospitalized before. Her family history is unremarkable.

Her examination revealed a tachypneic drowsy patient with a Glasgow coma scale (GCS) of 8. There was a photosensitive malar rash over her cheeks. Cardiac examination was normal with no murmurs and her lung auscultation revealed no abnormalities. Abdominal examination was normal and her musculoskeletal system examination was completely normal with no joints swellings or deformities. Her vitals at time of admission were as follows: PR: 110 bpm, RR: 24, BP: 80/50 mmHg and SaO2 of 81% on Room Air.

## 3 DIFFERENTIAL DIAGNOSIS AND INVESTIGATIONS

Up to this point, we have made a diagnosis of massive PE clinically on the basis of her modified well's score of 5.5 (HR > 100, Hemoptysis as well as no alternative diagnoses more likely than PE) in addition to the hemodynamic instability that our patient has suffered from which cannot be explained by any cardiac condition (given her normal cardiac exam and normal echocardiography excluding cardiac causes of her hemodynamic instability). A very high level of d‐dimer (10,000) has supported that diagnosis. Nevertheless, we were not able to do the CT pulmonary angiography scan of the chest due to financial and logistic causes as well as the patient's hemodynamic instability. A transthoracic echography was normal ruling out cardiac causes of possible presentation.

## 4 TREATMENT, OUTCOME, AND FOLLOW‐UP

When it comes to the treatment provided to our patient, we divided it into 2 phases. First, is the phase of stabilization in which the patient was resuscitated using saline infusion that resulted in raising her BP to 110/70 as well as an IV bolus of hydrocortisone 200 mg every 6 h. Second, is the maintenance phase in which the patient was commenced on hydroxychloroquine, meropenem, and pantoprazole. We also provided adequate analgesia in addition to zinc and vitamin C supplements.

Twenty four hours after admission, her fever improved, and the hemoptysis as well as the epigastric pain subsided. Nonetheless, she was still drowsy with a GCS of 11 and SaO2 of 90% off oxygen. Her vitals were as follows: PR = 110, BP = 100/60, RR = 28. We continued the same management plan but replacing hydrocortisone with methylprednisolone 500 mg, Clexane 6000 IU S.C every 12 h with quinine, doxycycline and also adding N‐acetylcysteine to the regimen. We also added vitamin D and Iron tablets.

On the third day of admission, calcium carbonate tabs 500 mg every 8 h were also added to aid with managing her hypocalcemia (Serum Ca = 7.1 mg/dl, Procalcitonin = 1.5). She also received a packed red cell for a few days during her admission resulting in her Hb level being raised from 6.9 to 10.6 gm/dl. Over the next few days and by the 8^th^ day of admission, her fever subsided and she gradually regained her normal mental status and level of consciousness. Her vitals were also stabilized and they were as follows: PR=75, BP = 120/70, RR = 14, and SaO2 of 98% off oxygen. Her nasal swab for COVID came back negative and she was then discharged on oral cefditoren, hydroxychloroquine, prednisone, iron tablets, omeprazole, and calcium carbonate. She has been followed up a month later without complications.

## 5 DISCUSSION

We report a case of a 16‐year‐old female patient with established systemic lupus erythematosus (SLE) diagnosis for 5 months and is currently on steroids presenting with hypocalcemia and pulmonary embolism diagnosed clinically in the setting of COVID‐19 infection.

In terms of infections, SLE increases the risk of infections, both common and opportunistic, with the lung being the most affected organ. It does so through impairing both arms of the immune system, the cellular and humoral immunity.^5^ COVID‐19 is not an exception. Coronavirus disease 2019 (COVID‐19) is a respiratory tract infection caused by severe acute respiratory syndrome coronavirus 2 (SARS‐CoV‐2), a coronavirus that spreads mainly through respiratory droplets.^6^ COVID‐19 infections, on the other hand, are also thought to increase the risk of autoimmune diseases through the production of autoantibodies and autoimmunity.^7^

With regard to thrombotic phenomenon, both COVID‐19 infection and SLE have been thought to cause such presentation. Complement activation in COVID‐19 can cause thrombotic microangiopathy that can mediate organ damage in severe cases, resembling a complement‐mediated thrombotic microangiopathy.^8^, ^9^ Some factors that may be implicated in thrombosis in SLE include immune response through the circulating complexes along with autoantibodies and the high state of inflammation that ensues during the disease activity.^10^

Hypocalcemia with COVID‐19 infection has been reported many times in the literature along with low lymphocytes and elevated LDH, liver enzymes, CK as well as CRP and is a frequent finding including a case of Italian patient with acute severe hypocalcemia. Mechanisms for such finding include a viral action requiring calcium for pathogenesis, malnutrition as well as abundance of unsaturated fatty acids that present in high levels during this viral infection, therefore, its severity can predict the outcome for patients, hence proper evaluation and management should be done.^11^

## 6 CONCLUSION

A combination of SLE with hypocalcemia and pulmonary embolism in the setting of COVID‐19 infection, while expected, has not been reported in the literature. This can be alarming since patients with SLE are already immunosuppressed with high risk of thrombosis. Adding the risk of thrombosis from COVID‐19, this can result in a massive pulmonary embolism as in our reported patient. Hence, in such patients, hypocalcemia should be thoroughly investigated, properly addressed, and adequately managed.

## CONFLICTS OF INTEREST

The authors report no conflict of interest of any sort.

## AUTHOR CONTRIBUTIONS

MYE and MMF served as the first author and the second author, collected the data, analyzed the results, and wrote the manuscript. MA and MA served as the third and the fourth authors, wrote the manuscript, revised the manuscript, and did editing. AI, KAH, and MSH served as the fifth, the sixth, and the seventh authors, collected, analyzed the data, wrote and revised the final draft. All authors read and approved the final manuscript.

## ETHICAL APPROVAL

Ethics approval was obtained from Gezira State Ministry of Health, and a written informed consent was taken from the patient.

## CONSENT

The authors have confirmed that patient consent has been signed and collected in accordance with the journal's patient consent policy.

## Data Availability

The data that support the findings of this study are available from the corresponding author upon reasonable request.
